# Study on dengue severity in diabetic and non-diabetic population of tertiary care hospital by assessing inflammatory indicators

**DOI:** 10.1016/j.amsu.2022.104710

**Published:** 2022-09-16

**Authors:** Ravinder Singh, Sanjay Goyal, Namita Aggarwal, Sanjana Mehta, Pratima Kumari, Varinder Singh, Hitesh Chopra, Talha Bin Emran

**Affiliations:** a Chitkara College of Pharmacy, Chitkara University, Rajpura, Punjab, 140401, India; b Government Medical College and Rajindra Hospital, Patiala, Punjab, India; c Department of Pharmacy, BGC Trust University Bangladesh, Chittagong, 4381, Bangladesh; d Department of Pharmacy, Faculty of Allied Health Sciences, Daffodil International University, Dhaka, 1207, Bangladesh

**Keywords:** Dengue, Diabetes, Endocan, Dengue haemorrhagic fever (DHF), Dengue shock syndrome, Hyperglycaemia

## Abstract

**Background:**

Dengue fever is a highly endemic tropical infectious disease that is quickly spreading over the world. Diabetes Mellitus has been linked to chronic inflammation. This present study was designed to compare the severity of dengue infection among diabetic and non-diabetic populations.

**Methods:**

A prospective observational study was conducted on 40 patients (20 diabetic and 20 non-diabetic) who suffered from dengue infection. The study involved the collection of data of the dengue patients includes patient's demographic details, medical condition as well as biochemical investigations.

**Results:**

Dengue-infected individuals with diabetes showed greater CRP, Endocan levels, IL-8 and Perfusion Index than those without diabetes (CRP; 35.308 ± 1.32 vs. 18.6365 ± 0.64) mg/dl (*p≤* 0.001) (Endocan 42.316 ± 1.46vs. 32.839 ± 0.33), ng/dl (*p≤* 0.001), (142.98 ± 1.05 vs 103.69 ± 0.64) (*p* ≤ 0.001) and (3.695 ± 0.18 vs. 1.98 ± 0.08) (*p* ≤ 0.001) respectively.

**Conclusion:**

In conclusion the results indicate that prognosis of DHF grade II with diabetes mellitus tends to be more prone to bleeding disorder and can result into morbidity and mortality considering by triggering of the various inflammatory cascade resulting in hyperglycaemia and poor glycemic control.

## Highlights

• Dengue fever is a highly endemic tropical infectious disease.
• Present study aims compares the severity of dengue infection among diabetic and non-diabetic populations.
• The study involved the collection of data of the dengue patients includes patient's demographic details.
• The results indicate that prognosis of DHF grade II with diabetes mellitus tends to be more prone to bleeding disorder.

## 1 Introduction

Dengue fever is a well-known vector borne viral disease that is widespread in tropical and subtropical areas of the world and is associated with higher incidence and mortality rate [1]. Globalization, rising air travel, and unintended urbanisation have all contributed to a rise in infection rates, as well as helping dengue to spread geographically and demographically [2]. Dengue viruses cause a variety of clinical signs, ranging from asymptomatic infection to a self-limited febrile illness to severe dengue, which is characterized by increased capillary permeability and shock [3]. Pathologically, dengue is characterised by high levels of inflammatory markers like CRP, endocan and IL-8. Thus, the dengue could be more fatal in patients that have co-morbid conditions of inflammation such as in diabetes [4]. Over the decades numerous research indicated close link between diabetes and inflammation. Hyperglycaemia alters the morphological and physiological integrity of the endothelium leading to a chronic inflammatory state produced by T-lymphocyte activation and production of proinflammatory cytokines such as gamma interferon (IFN) and TNF [5,6]. These cytokines are known to play an important role in one of the primary symptoms of complex dengue fever. Further, DM has been discovered to have negative effects on the immune system, such as reduced chemotaxis, leukocyte adhesion, and infection phagocytosis making a person more susceptible to infection [[7], [8], [9], [10], [11]].

Since a person with diabetes has weak immunity, fragile blood vessels and a higher risk of haemorrhage, symptoms of dengue tend to worsen in diabetics. Because the prevalence of both diseases is increasing, it is critical to understand the relationship between severe dengue and diabetes mellitus [12]. Therefore, this study is aimed to gain the better picture for understanding the inflammatory events in diabetic and non-diabetic population with dengue infection.

## 2 Materials and methods

### 2.1 Study design

**Fig. 1:**
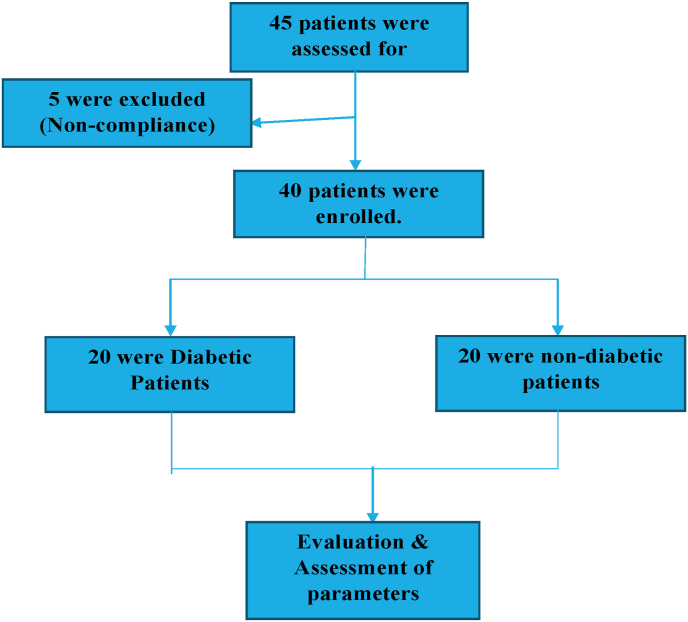
Flow diagram of screening.

Patients were enrolled in this prospective observational study from a tertiary care hospital of North India between October and November 2021. The study was conducted as per the declaration of Helsinki principle. This study comprised 40 patients who had been diagnosed with dengue fever in which 20 patients were diabetic and 20 patients were non-diabetic (Fig. 1).

### 2.2 Participants

Eligibility criteria for enrolment were: Patient ages between 15 and 65 years, patient with confirmed dengue diagnosis and patient with or without diabetes since 5 years. Exclusion criteria were: Pregnant females, patients with autoimmune or chronic infectious disease and patients with haematological disorders and neoplasia were excluded.

For each patient, we gathered the following information in the case record form: age, gender, medical history (diabetes and duration), height, and weight. Blood samples were taken from these patients on the first day. The haematological biochemical analysis includes white blood cell (WBC) count, hematocrit, platelet count, blood glucose, serum creatinine, liver function tests (SGOT and SGPT), serum albumin, total protein, and glycated haemoglobin (HbA1c for diabetic patients only). Blood pressure of all the patient's were taken and included in the analysis.

Using an ELISA test kit and laboratory studies, blood samples were taken and centrifuged to provide serum for the measurement of inflammatory biomarkers such as C - reactive protein, Serum Endocan, Interlukein 8 and Perfusion Index.

### 2.3 Ethical approval statement

The study was aimed to define the impact of dengue infection in Diabetes Mellitus/Non- Diabetic population of North India by assessing various inflammatory markers. The study has been approved by the institutional ethical committee Chitkara University Punjab approval number: EC/NEW/INST/2021/531/76 and was performed in accordance with the declaration of Helsinki and the code of Good Clinical practice.

### 2.4 Statistical analysis

All data was expressed as mean ± square error of mean (SEM). Analyses of the data obtained in the study were made using GraphPad Prism software version 9.3.1. The variables were compared using the student *t*-test between diabetic and non-diabetic patients. The Mann-Whitney *U* test was used to compare the differences in endocan, CRP and perfusion index respectively between both the groups. The correlation between inflammatory markers and HbA1c data was assessed by Pearson coefficient correlation test. A value of *p* < 0.05 was considered statistically significant.

## 3 Results

The socio-demographics, symptoms, bleeding type and laboratory characteristics of study population (n = 40) from which (n = 20) were suffering with dengue with diabetes and (n = 20) were suffering with dengue without diabetes are shown in Table 1. The average age of patients with dengue with diabetes was 55.45 ± 2.71 and that with dengue without diabetes was 57.55 ± 3.20. Females were more in number as compared to males of those suffering with dengue with diabetes and those without diabetes also. The bleeding sites were also assessed where those with skin (petechiae) damaged were more in people suffering with dengue with diabetes as compared to other study group while those with gastrointestinal system (melena) and mucous membrane (epistaxis) being the bleeding sites were more in patients with dengue without diabetes and were statistically significant (*p* ≤ 0.05).

**Table 1:** Clinical characteristics of dengue-infected patients in study population.

Variables	Non-diabetic	Diabetic	*p-value*
Number	20	20	–
**Demographics**			
Age	57.55 ± 3.20	55.45 ± 2.71	0.619
30–45%	7 (35%)	8 (40%)	–
46–60%	11 (55%)	9 (45%)	–
61–75%	2 (10%)	3 (15%)	–
Male	48.25 ± 2.64	54.09 ± 2.64	0.41
Female	60.25 ± 2.60	62.88 ± 2.46	0.48
**Symptoms**			
Fever (%)	15 (75%)	18 (9%)	0.40
Arthralgia (%)	14 (70%)	16 (80%)	0.71
Myalgia (%)	12 (60%)	15 (75%)	0.50
**Site of Bleeding**			
Petechiae (%)	12 (60%)	16 (80%)*	0.30
Malena (%)	4 (20%)	3 (15%)	1.00
epistaxis (%)	4 (20%)	1 (5%)	0.3
**Laboratory Results**			
HbA1C (%)	5.27 ± 0.03	6.39 ± 0.09*	<0.001
SGPT (units/l)	104.55 ± 8.58	139.3 ± 12.21*	0.02
SGOT (units/l)	149.1 ± 8.65	180.2 ± 11.28*	0.03
S. Creatinine (mg/dl)	0.18 ± 0.05	1.03 ± 0.36*	0.02
S. Albumin (g/dl)	1.24 ± 0.14	0.69 ± 0.07*	<0.001
Platelet Count ( × 10^3^ cells/μL)	58.36 ± 1.634	49.758 ± 3.349*	0.02

The clinical characteristics such as blood glucose (HbA1C), SGPT, SGOT and serum creatinine were statistically significant in individuals with dengue and diabetes (*p* ≤ 0.05) while serum albumin and platelet count levels were also found to be statistically significant (*p* ≤ 0.05) but lower as compared to the other study group as shown in Table 1.

**Fig. 2:**
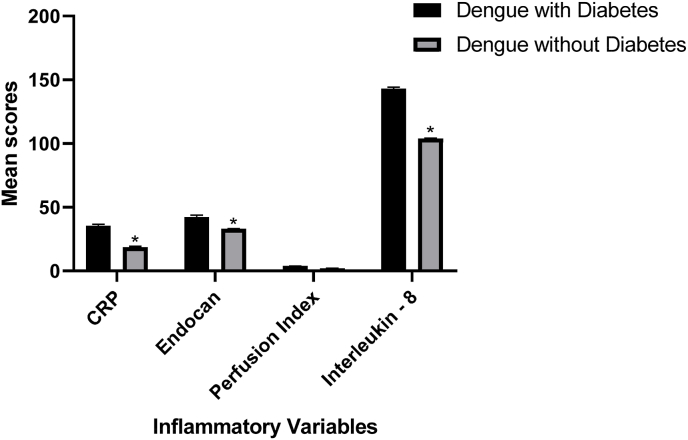
Correlation values of inflammatory variables of dengue patients with and without diabetes.

The inflammatory variables (C-reactive protein, Endocan, Interleukin-8 and Perfusion Index) were also checked in both the groups i.e., dengue with diabetes and dengue without diabetes. The entire three inflammatory mediators were found to be more in subjects suffering with dengue and diabetes as compared to those with dengue without diabetes and were also found to statistically significant (*p* ≤ 0.05) (Table 2 and Fig. 2).

**Table 2:** Comparison of dengue patients with diabetes and without diabetes.

Inflammatory variables	Dengue with diabetes	Dengue without diabetes	*p value*
CRP (mg/dl)	35.308 ± 1.32	18.6365 ± 0.64*	<0.0001
Endocan (ng/dl)	42.316 ± 1.46	32.839 ± 0.33*	<0.0001
IL-8 (pg/ml)	142.98 ± 1.05	103.69 ± 0.64*	<0.0001
Perfusion Index (%)	3.695 ± 0.18	1.98 ± 0.08	<0.0001

**Fig. 3:**
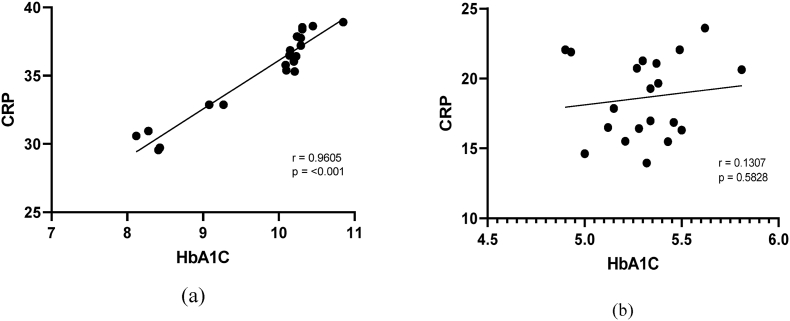
Correlation between HbA1C and CRP in patients (a) with diabetes and(b) without diabetes.

**Fig. 4:**
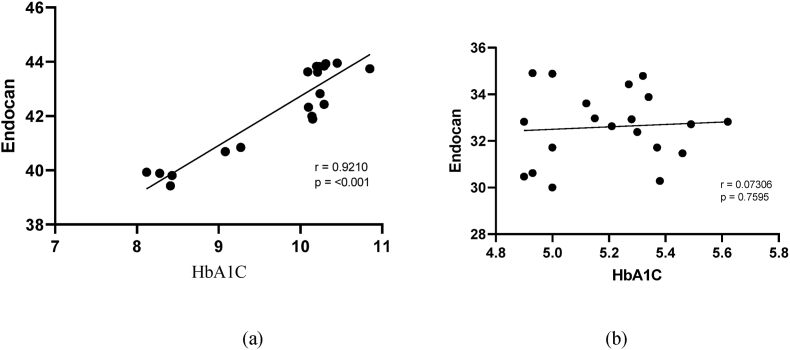
Correlation between HbA1C and Serum Endocan in patients (a) with diabetes and(b) without diabetes.

**Fig. 5:**
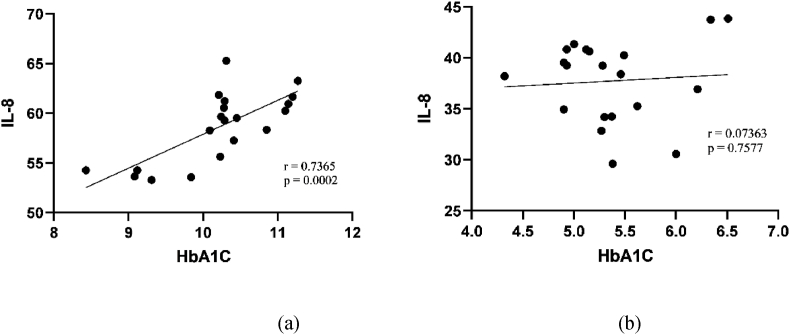
Correlation between HbA1C and IL-8 in patients with (a) diabetes and (b) without diabetes.

**Fig. 6:**
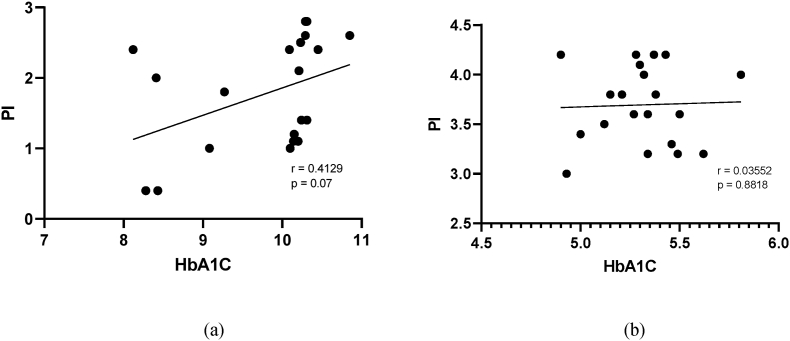
Correlation between HbA1C and PI in patients (a) with diabetes and (b) without diabetes.

The Univariate Pearson correlation (Table 3) depict for the inflammatory variables and both groups, i.e. dengue with diabetes and dengue without diabetes. Inflammatory components (CRP, endocan and perfusion index) exhibited a statistically significant (*p* ≤ 0.05) positive correlation in subjects with dengue with diabetes. Serum CRP and Endocan levels showed strong while perfusion index showed intermittent positive relationship with the dengue with diabetes group. Furthermore, in the other study group, inflammatory markers (CRP, Endocan, IL-8, and perfusion index) exhibited no linear relationship or nearby no correlation along with statistically non-significant subjects suffering with dengue without diabetes (Fig. 3, Fig. 4, Fig. 5, Fig. 6).

**Table 3:** Correlation values of inflammatory variables of dengue patients with and without diabetes.

Inflammatory variables		Dengue	
		With diabetes	Without diabetes
CRP	R	0.9605*	0.1307
	*p-*value	<0.001	0.5828
Endocan	R	0.9210*	0.07306
	*p-*value	<0.001	0.7595
IL-8	R	0.7365*	0.07363
	*p-*value	0.0002	0.7577
Perfusion Index	R	0.4129	0.03552
	*p-*value	0.07	0.8818

## 4 Discussion

The prevalence and frequency of diabetes and dengue is increasing internationally. However, it is critical to determine the relationship between dengue and diabetes mellitus. Moreover, if severity of dengue keeps on rising in uncontrolled or poorly controlled diabetes patients then it would result into higher morbidity and mortality in comparison to diabetic and non-diabetic population [[13], [14], [15]]. Diabetic patients showed a larger proportion of increased inflammatory markers and lower platelet counts in the current study, suggesting that diabetes may be a predisposing factor towards more severe dengue infection. Despite the fact that multiple studies have revealed that diabetes mellitus can cause immunological and endothelial dysfunction, the pathophysiology behind diabetes leading to Dengue Haemorrhagic Fever (DHF) is still unknown [16].

In this study, 40 patients were enrolled as per the inclusion and exclusion criteria; further patients were classified randomly in two groups i.e., diabetic and non-diabetic (20 patients per group). Among the study population, the maximum no. of patients with diabetes was found to be in 46–60 years age group (55%), followed by 30–45 years age group (35%), and in 61–75 years age group (10%). Similarly, the maximum no. of non-diabetic patients was found to be in 46–60 years age group (45%), followed by 30–45 years age group (40%), and in 61–75 years age group (15%).

In the previous study, Pang et al., performed the retrospective case-control study which showed that adult dengue patients with diabetes were at greater risk of developing DHF during epidemic of predominantly serotype with adjusted odd ratio of 1.78 (1.06–2.97). Multivariate logistic regression was used to assess the association between DHF and co-morbidities [17]. In 2006, Chen et al., indicated diabetes can be a risk factor for mortality in adult DHF patients by demonstrating that serum levels of inflammatory markers(IL-6, IL-10 and CRP) was increased and positively correlated with the disease severity [13]. The current study also reveals that serum levels of inflammatory markers CRP were tremendously increased in the patients with diabetes (35.308 ± 1.32) mg/dl than in non-diabetic patients (18.6365 ± 0.64) mg/dl.

Raj et al., performed a case control study over a period of 12 months and concluded that Hyperglycaemia is linked to a worse outcome in diabetic dengue patients, regardless of the severity of other symptoms such thrombocytopenia. The rising global frequency of both dengue fever and diabetes underpins the need for more research in this area. The necessity of the hour is to confirm dengue infection and recognise its consequences in diabetic individuals as soon as possible. In the study, we have revealed that there is positive correlation between higher serum HbA1C levels and inflammatory markers (CRP, Endocan and PI) on the basis of correlation analysis [18].

The occurrence of hyperglycaemia in diabetic patients with dengue necessitates better glycemic control monitoring to reduce the risk of a severe dengue clinical presentation [19].According to Lee and colleagues, patients with poor glycemic control (HbA1C>7%), with or without an additional co morbidity, had a higher risk of developing dengue haemorrhagic fever (DHF) and dengue shock syndrome (DSS) than those with diabetes who had adequate glycemic control and no additional co morbidity [20].

This is the first study from North India to compare the inflammatory cascade (CRP, Endocan and Perfusion Index) of dengue patients associated with and without diabetes. In this investigation, we discovered that dengue patients with diabetes had lower serum albumin levels (0.69 ± 0.07) g/dl and platelet counts (49.758 ± 3.349) × 10^3^ cells/μL than those without diabetes having serum albumin levels (1.24 ± 0.14) g/dl and platelet counts (58.36 ± 1.634) × 10^3^ cells/μL.

The inflammatory variables were significantly increased in dengue patients with diabetes i.e., CRP (35.308 ± 1.32) mg/dl, Endocan(42.316 ± 1.46) ng/dl, IL-8 (142.98 ± 1.05) pg/ml and Perfusion Index (3.695 ± 0.18) %in comparison with dengue patients without diabetes, CRP (18.6365 ± 0.64) mg/dl, Endocan (32.839 ± 0.33) ng/dl, IL-8 (103.69 ± 0.64) pg/ml and Perfusion Index (1.98 ± 0.08) %.

Increased levels of CRP and Endocan trigger endothelial dysfunction which may be because of the biological mechanism that leads to the severity of dengue fever in diabetic subjects, by increasing the intrinsic permeability of the endothelial surface resulting with dengue complications in the diabetic population.

In the current study, on finding the correlation between inflammatory variables (CRP, Endocan, IL-8 and Perfusion Index) and glycated haemoglobin (HbA1C), a positive correlation was indicated in dengue patients with diabetes whereas linear correlation was found in case of dengue patients without diabetes. Hence it is required to control the diabetes in the patients for better outcomes and further control the spread of dengue infection with more national eradicated programme by government authorities (National Vector Borne Disease Control Programme).

## Provenance and peer review

Not commissioned, externally peer reviewed.

## Ethical approval

This article does not require any human/animal subjects to acquire such approval.

## Sources of funding

This study received no specific grant from any funding agency in the public, commercial, or not-for-profit sectors.

## Author contributions

Ravinder Singh: Conceptualization, Data curation, Writing-Original draft preparation, Writing- Reviewing and Editing. Sanjay Goyal: Conceptualization, Data curation, Writing-Original draft preparation, Writing- Reviewing and Editing. Namita Aggarwal: Data curation, Writing-Original draft preparation, Writing- Reviewing and Editing. Sanjana Mehta: Data curation, Writing-Original draft preparation, Writing- Reviewing and Editing. Pratima Kumari: Writing- Reviewing and Editing. Varinder Singh: Writing- Reviewing and Editing. Hitesh Chopra: Data curation, Writing-Original draft preparation, Writing- Reviewing and Editing. Talha Bin Emran: Conceptualization, Writing-Reviewing and Editing, Visualization.

## Registration of research studies

1. Name of the registry: EC/NEW/INST/2021/531/76.
2. Unique Identifying number or registration ID: EC/NEW/INST/2021/531/76.
3. Hyperlink to your specific registration (must be publicly accessible and will be checked): Not applicable.

## Guarantor

Talha Bin Emran, Ph.D., Associate Professor, Department of Pharmacy, BGC Trust University Bangladesh, Chittagong 4381, Bangladesh. T: +88-030-3356193, Fax: +88-031-2550224, Cell: +88-01819-942214. https://orcid.org/0000-0003-3188-2272. E-mail: talhabmb@bgctub.ac.bd.

## Consent

Not applicable.

## Declaration of competing interest

All authors report no conflicts of interest relevant to this article.
